# Mentally-Deficient Children: Their Treatment and Training

**Published:** 1896-03

**Authors:** 


**Mentally-Deficient Children: their Treatment and Training.** By G. E. Shuttleworth, M.D. Pp. xiv., 140. London: H. K. Lewis. 1895.

The author has given in moderate compass a good account of his subject, with which, by long experience, he is well qualified to deal. The medical practitioner is often called upon to advise the parents as to what extent of mental development may be expected in a mentally deficient child, and as to what treatment and training should be adopted. We think this book will be of real service in giving succinctly the information necessary to arrive at a right conclusion. It opens with a short historical retrospect as to what has hitherto been successfully accomplished in the way of training at institutions for idiots and feeble-minded children. After an account of the pathological changes found in such cases, the author discusses the questions of etiology, prognosis, and diagnosis in the different forms of feeble-mindedness. In the remaining chapters the treatment, medical, moral, and educational, is fully considered, and the best mode of training described. These chapters contain much sound practical advice; and as the work is well and clearly written, and the illustrations well selected, we think that it will be found useful.

